# Global Burden of *Cyclospora cayetanensis* Infection and Associated Risk Factors in People Living with HIV and/or AIDS

**DOI:** 10.3390/v14061279

**Published:** 2022-06-12

**Authors:** Saba Ramezanzadeh, Apostolos Beloukas, Abdol Sattar Pagheh, Mohammad Taghi Rahimi, Seyed Abdollah Hosseini, Sonia M. Rodrigues Oliveira, Maria de Lourdes Pereira, Ehsan Ahmadpour

**Affiliations:** 1 Infectious and Tropical Diseases Research Center, Tabriz University of Medical Sciences, Tabriz 51666-14766, Iran; saba.ra94@yahoo.com; 2 Department of Parasitology and Mycology, Faculty of Medicine, Tabriz University of Medical Sciences, Tabriz 51666-14766, Iran; 3 National AIDS Reference Center of Southern Greece, Department of Public Health Policy, University of West Attica, 11521 Athens, Greece; abeloukas@uniwa.gr; 4 Molecular Microbiology & Immunology Lab, Department of Biomedical Sciences, University of West Attica, 12243 Athens, Greece; 5 Infectious Diseases Research Center, Birjand University of Medical Sciences, Birjand 97178-53577, Iran; satar2011@gmail.com; 6 Center for Health Related Social and Behavioral Sciences Research, Shahroud University of Medical Sciences, Shahroud 36147-73955, Iran; rahimimt@gmail.com; 7 Department of Parasitology and Mycology, Faculty of Medicine, Mazandaran University of Medical Sciences, Sari 33971-48157, Iran; hosseini4030@gmail.com; 8 CICECO-Aveiro Institute of Materials, University of Aveiro, 3810-193 Aveiro, Portugal; sonia.oliveira@ua.pt; 9 Hunter Medical Research Institute, New Lambton, NSW 2305, Australia; 10 Department of Medical Sciences, University of Aveiro, 3810-193 Aveiro, Portugal; 11 Immunology Research Center, Tabriz University of Medical Sciences, Tabriz 51666-14766, Iran

**Keywords:** *Cyclospora cayetanensis*, HIV, AIDS, protozoan parasite, prevalence, meta-analysis

## Abstract

*Cyclospora cayetanensis* infections remain one of the most common protozoan opportunistic causes of gastrointestinal diseases and diarrhea among people living with HIV and/or AIDS (PLWHA). This study was conducted to provide a summary of the evidence on the global burden of *C. cayetanensis* infection and associated risk factors among PLWHA. *Scopus, PubMed, Science Direct*, and *EMBASE* were searched up to February 2022. All original peer-reviewed original research articles were considered, including descriptive and cross-sectional studies describing *C. cayetanensis* in PLWHA. Incoherence and heterogeneity between studies were quantified by I index and Cochran’s Q test. Publication and population bias were assessed with funnel plots and Egger’s asymmetry regression test. All statistical analyses were performed using StatsDirect. The pooled prevalence of *C. cayetanensis* infection among PLWHA was 3.89% (95% CI, 2.62–5.40). The highest prevalence found in South America was 7.87% and the lowest in Asia 2.77%. In addition, the prevalence of *C. cayetanensis* was higher in PLWHA compared to healthy individuals. There was a relationship between a higher *C. cayetanensis* prevalence in PLWHA with a CD4 cell count below 200 cells/mL and people with diarrhea. The results show that PLWHA are more vulnerable to *C. cayetanensis* infection and emphasizes the need to implement the screening and prophylaxis tailored to the local context. Owing to the serious and significant clinical manifestations of the parasite, an early identification of seropositivity is recommended to initiate prophylaxis between PLWHA with a CD4 count ≤200 cells/mL and PLWHA who do not receive antiviral therapy.

## 1. Introduction

The upper and lower gastrointestinal (GI) tract plays a critical role in both the clinical manifestations and pathogenesis of HIV infection. People living with HIV and/or AIDS (PLWHA) are more vulnerable to a variety of opportunistic infections, including gastrointestinal parasitosis [1,2]. Recently, the UNAIDS report estimated that there would be over thirty-eight million PLWHA at the end of 2020 [3]. More than half of immunocompromised PLWHA experience diarrheas that can cause significant morbidity, contributing negatively to the quality of life and to adherence to antiretroviral therapy (ART). This may be due to a multitude of etiologies from infectious pathogens to malignancy to medications [4,5,6]. Over the past decade, due to the unprecedented increase in the use of ART, the incidence of diarrhea from opportunistic infections has decreased [6]; however, it remains a remarkable threat. Opportunistic infectious pathogens that cause diarrhea in PLWHA span a variety of bacteria, fungi, viruses, and parasites. The latter include *Toxoplasma*, *Cryptosporidium*, *Cystoisospora,* and Cyclospora genera that cause moderate to severe diseases [7,8]. Parasitic infections and HIV interact, and parasitic infections may activate the proliferation of HIV and accelerate the progression of the disease from HIV to AIDS. PLWHA with CD4 counts below 200 cells/mL are more prone to GI parasitic infections and to develop disease complications [9]. Apart from diarrhea, GI parasitic infections in immunocompromised PLWHA can cause symptoms such as abdominal pain, fever and chills, muscle aches, eosinophilia, frequent urination and hematuria, clinical manifestations of the central nervous system, weight loss, and transient pneumonia and, in the case of advanced HIV disease, can lead to death [5,10]. *Cyclospora cayetanensis* is a microscopic food- and waterborne coccidian parasite that is endemic in tropical and subtropical regions [11,12,13,14]. *C. cayetanensis* infection occurs by ingesting of sporulated oocysts, which are the infective form of the parasite. An infected person sheds unsporulated (immature, non-infective) *Cyclospora* oocysts in the feces. Oocysts must be sporulated at a temperature of 25–30 °C for at least 1–2 weeks to become infective. Therefore, direct person-to-person transmission is almost impossible, as is transmission via ingestion of newly contaminated food or water. It is thought that the main cause of the spread of *C. cayetanensis* infection is by ingesting sporulated oocysts from contaminated water and food and lack of hygiene. Clinical manifestations are limited in immunocompetent people but cause chronic watery diarrhea and severe GI damage in immunocompromised patients [15]. There are very limited data on the prevalence of this parasite in PLWHA, and due to the COVID-19 pandemic, we are seeing an increase in the number of immunocompromised patients [16]. Thus, further research will be required to fill this gap of knowledge. Therefore, we conducted a systematic review study to assess the burden of *C. cayetanensis* parasitosis in PLWHA to implement better prevention and treatment strategies.

## 2. Materials and Methods

### 2.1. Search Strategy and Selection Criteria

This systematic review was conducted according to the principles outlined in the PRISMA statement (Preferred Reporting Items for Systematic and Meta-Analysis) and PRISMA-P checklist [17]. Search methods attempted to identify all relevant studies regardless of language, date of publication, or publication status. Two independent investigators (A.S.P., S.R.) systematically searched electronic databases, including *PubMed, ProQuest, Scopus, Science Direct*, and *Google Scholar*. The final search was conducted up to 28 February 2022. Keywords used for the searches were Cyclospora, *Cyclospora cayetanensis*, Cyclosporiasis, intestinal parasite, immunocompromised patients, HIV, AIDS, epidemiology, and prevalence.

Studies were included if they met the following criteria: (1) papers published in the English language, (2) articles presenting people living with HIV and/or AIDS, and (3) articles showing the age of patients and the geographical area. We also excluded studies if they were case reports, letter to the editor, reviews, animal studies, or duplicates. After removing duplicates using the Endnote program (www.endnote.com, accessed on 14 February 2022), titles and abstracts of unique papers identified in the search results were independently screened by two authors (A.S.P., S.R.) according to inclusion and exclusion criteria. Full texts were retrieved for all citations marked as “included”. Where appropriate, multiple reports on the same study were identified and merged. Disagreements were resolved by discussion or with the assistance of a third author (E.R.). We also used the PRISMA Flow Diagram. Authors were contacted where data were unclear. Individual patient-level data were sought.

### 2.2. Assessment of Risk of Bias and Quality in the Included Studies

Two authors independently assessed the quality of the studies using the JBI (Joanna Briggs Institute) checklist [18]. These tools rate the quality of selection, measurement, and comparability and give a score to the studies (maximum of 9). This tool comprised nine items with four options: “yes”, “no”, “unclear”, and “not applicable”. “Yes” answers were used to calculate the final score of each article.

### 2.3. Data Extraction and Analysis

A study-level data extraction table was designed, piloted, and modified appropriately using Microsoft Excel (Microsoft Office^®^, 2019 version). The data extraction form included the following fields: year of publication, region, study design, sample sizes, gender, number of people with diarrhea, CD4 counts of patients, prevalence of *C. cayetanensis*, diagnostic method, interfering factor, and HAART. Duplicate data were noted and excluded.

### 2.4. Meta-Analysis

The primary aim was to assess the global prevalence of *Cyclospora parasitosis* in PLWHA. The statistical heterogeneity between studies was assessed using Cochran’s and I^2^ tests. For meta-analysis purposes, a random-effects model was used. The meta-analysis was completed with the trial version of the StatsDirect statistical software (www.statsdirect.com, accessed on 7 March 2022). A forest plot was applied to show the heterogeneity between studies. It showed proportions of individual studies and total prevalence of *C. cayetanensis*.

## 3. Result

### 3.1. Search Results

**Figure 1:**
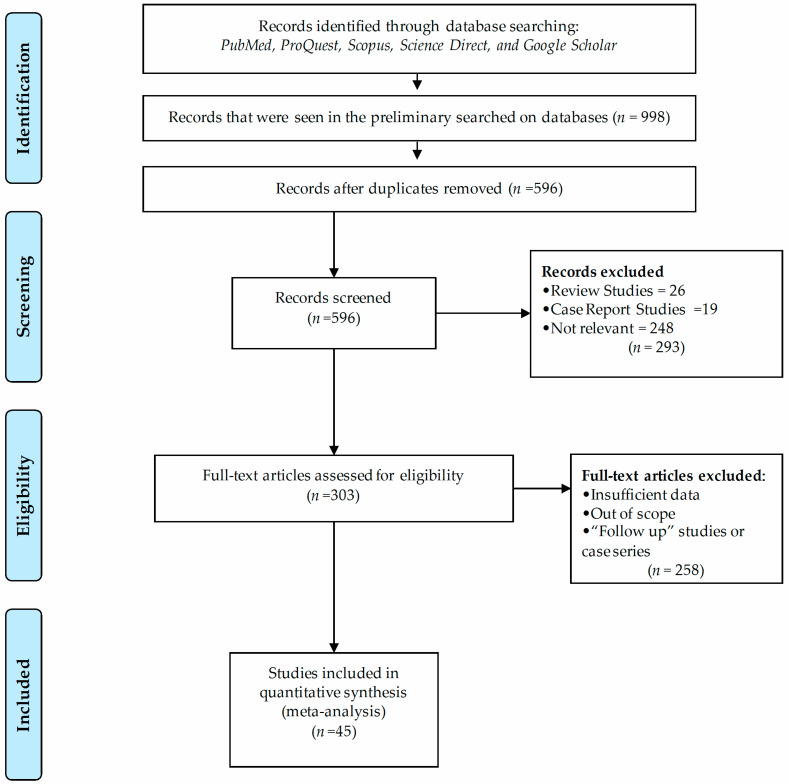
Search strategy and study selection process indicating numbers of studies excluded or included using PRISMA flow diagram.

Our preliminary search of five scientific databases yielded 998 records. From that, 402 were excluded as duplicate records. Of the 596 remaining records, 293 articles were excluded after review of titles and abstracts. These included 6 review articles, 19 case reports, and 248 irrelevant articles. Then, the full text of 303 articles was evaluated, and 258 studies did not meet our inclusion criteria. Finally, we retrieved 45 full texts to assess the eligibility for inclusion, and these were included in the systematic review and meta-analysis. A PRISMA diagram of the screening process is depicted in Figure 1.

### 3.2. Characteristics of Studies

The quality of the studies was assessed using the JBI critical appraisal checklist. None of the studies assessed for quality by the JBI checklist were excluded due to lack of merit. As a result of the reviews, of the 45 articles: two articles received 3 points, four articles received 4 points, twelve articles received 5 points, thirteen articles received 6 points, eight articles received 7 points, three articles received 8 points, and four articles received 9 points. In total, the score was 6 (moderate quality) (Table 1). In total, 9310 PLWHA were included in our study. The identified studies were conducted in 21 countries across four continents. Studies selected included reports from North America (20%, 9/45), South America (6.6%, 3/45), Asia (53.3%, 24/45), and Africa (20%, 9/45). All of these were conducted between 1994 and 2022.

Q1: Was the sample frame appropriate to address the target population?

Q2: Were study participants sampled in an appropriate way?

Q3: Was the sample size adequate?

Q4: Were the study subjects and the setting described in detail?

Q5: Was the data analysis conducted with sufficient coverage of the identified sample?

Q6: Were valid methods used for the identification of the condition?

Q7: Was the condition measured in a standard, reliable way for all participants?

Q8: Was there appropriate statistical analysis?

Q9: Was the response rate adequate, and if not, was the low response rate managed appropriately?

Europe and Oceania had no study meeting the inclusion criteria. Study types included cross-sectional (62.2%, 28/45), case-control studies (20%, 9/45), retrospective (13.3%, 6/45), and cohort (4.4%, 2/45) studies. In all included studies (100%, 45/45), staining was used for the diagnosis of *C. cayetanensis* infection and, in four included studies (8.8%, 4/45), molecular methods (Table 2). A number of studies used several methods at the same time to confirm the presence of *C. cayetanensis* [7,19,20,21]. The overall male-to-female ratio was 59.35% to 40.64% (M:F = 1.46:1) among all PLWHA. In total, 44.7% (485/1085) of PLWHA had diagnosed diarrhea, 34% (1130/3317) had CD4 counts <200 cells/mL, and 74.5% (444/596) were on highly active antiretroviral therapy (HAART), while 55.36% (821/1483) received antibiotics.

### 3.3. Statistical Analysis

**Figure 2:**
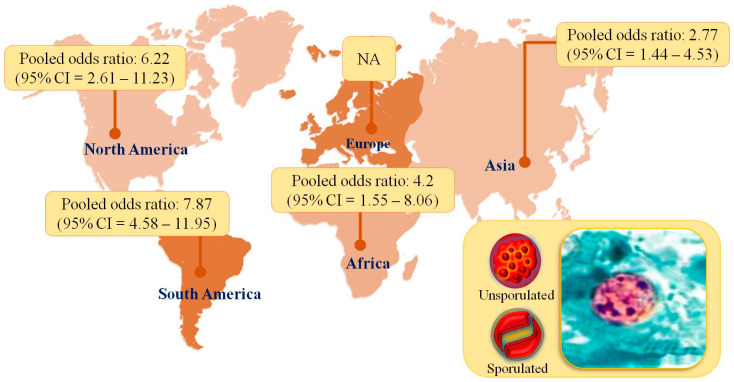
Pooled prevalence of *C. cayetanensis* in HIV-infected patients in different continents.

**Figure 3:**
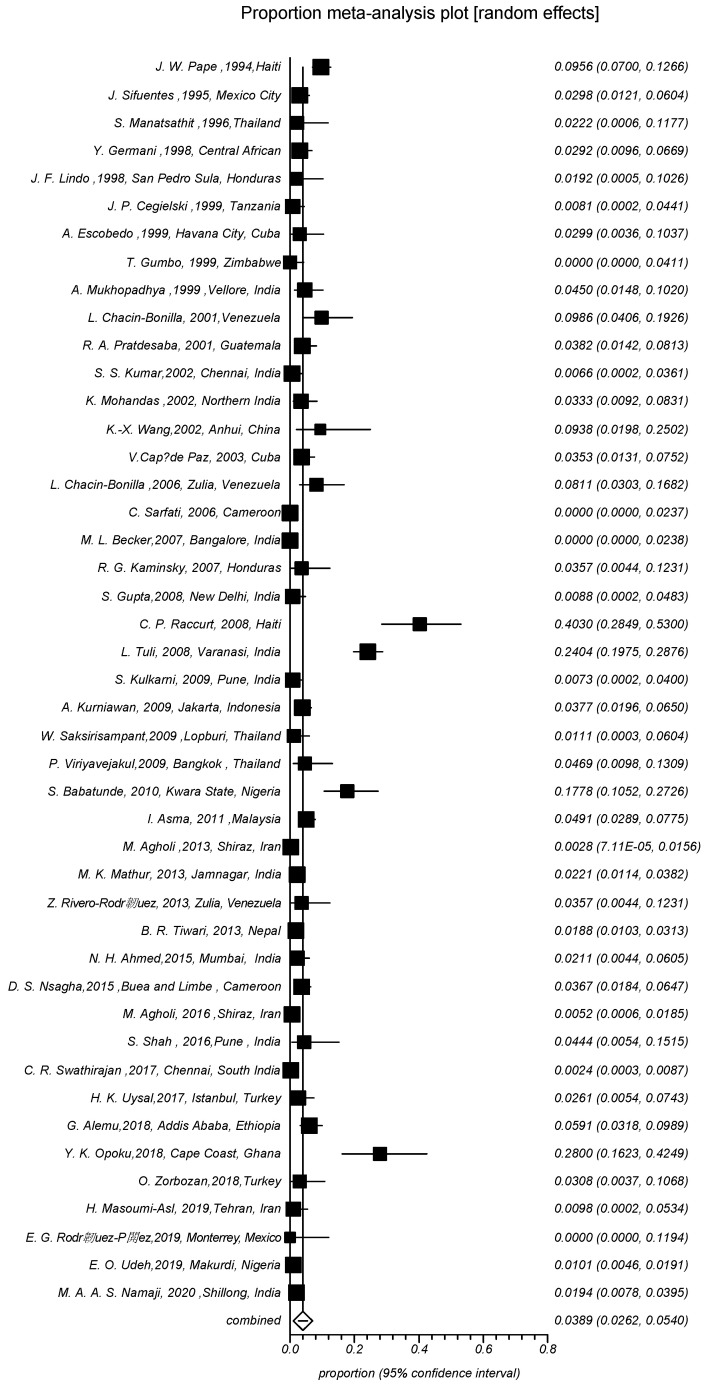
Random-effects meta-analysis of *C. cayetanensis* infection in people living with HIV and/or AIDS (PLWHA).

**Figure 4:**
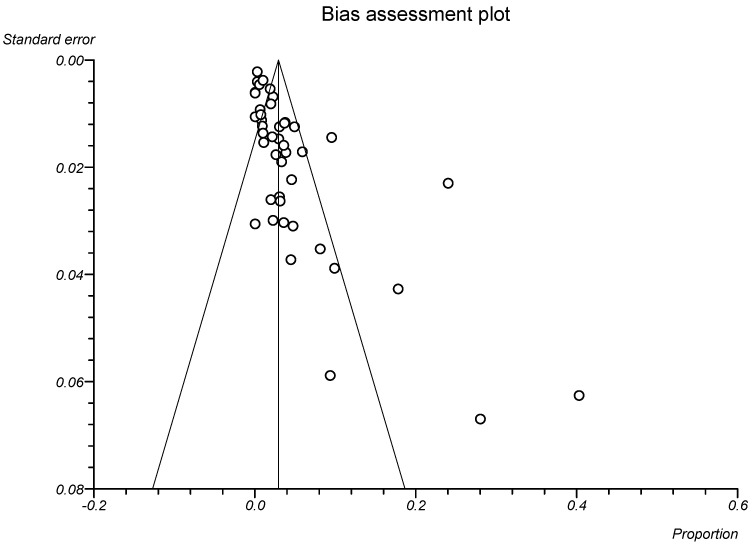
Bias assessment plot displaying the prevalence estimate of prevalence of *C. cayetanensis* infection in people living with HIV and/or AIDS.

The estimated global prevalence of Cyclospora parasitosis in PLWHA ranged from 0.0% to 40.3%. Of 9310 samples, 364 were infected with *C. cayetanensis*. The estimated global pooled prevalence of *C. cayetanensis* infection in PLWHA using the random effects model for meta-analysis was 3.89% (95% CI, 2.62–5.40(. The prevalence of the parasite in North America, South America, Asia, and Africa was estimated at 6.22% (95% CI 2.61–11.23, 94/1283), 7.87% (95% CI, 4.58–11.95, 15/201), 2.77% (95% CI, 1.44–4.53, 576/186), and 4.2% (95% CI, 1.55–8.06, 69/2088), respectively. The geographic distribution of *C. cayetanensis* infection in PLWHA is shown in Figure 2. The pooled prevalence of *C. cayetanensis* infection in men compared to women (OR = 1.72, 95% CI, 0.79–3.73, *p* = 0.1647) was also estimated. Furthermore, the pooled prevalence of *C. cayetanensis* in PLWHA with diarrhea compared with/without diarrhea was estimated (OR = 3.23, 95% CI, 1.38–7.54, *p* = 0.0066). The pooled prevalence of *C. cayetanensis* in patients with a CD4 counts <200 cells/mL compared to patients with a CD4 count of more than 200 cells/mL was estimated (OR = 4.07, 95% CI, 1.37–12.12, *p* = 0.0115). Moreover, the pooled prevalence of *C. cayetanensis* in patients who did not receive HAART compared with patients with HAART was estimated (OR = 2.07, 95% CI, 0.29–14.81, *p* = 0.4668) (Table 3), and the pooled prevalence of *C. cayetanensis* in PLWHA compared to people without HIV was estimated (OR = 4.36, 95% CI, 2–9.48, *p* = 0.0002). There was a broad difference in the prevalence rate between various studies. Furthermore, the Cochran’s Q statistic was (Q = 506.06, df = 44, *p* < 0.000, I^2^ = 91.3%, 95% CI, 89.6%–92.6%) (Figure 3). Inspection of the bias assessment plot showed publication bias, and a statistically significant Egger’s regression suggests the possibility of publication bias (Figure 4).

## 4. Discussion

This systematic review and meta-analysis provide comprehensive data on the global prevalence of *C. cayetanensis* in PLWHA. Our findings highlight the high global burden on PLWHA. The global pooled prevalence of *C. cayetanensis* was 3.89% and was significantly higher in people with diarrhea, OR = 3.23 (95% CI, 1.38–7.54). There were contradictory studies regarding to the prevalence of the parasite, especially in immunocompromised individuals. A study by Chacín-Bonilla reported the rate of *C. cayetanensis* infection up to 2010 [11], with a prevalence varying from 0% to 13% in 47,642 apparently immunocompetent individuals, most with diarrhea. Furthermore, the prevalence rate in matched asymptomatic controls varied from 0% to 4.2% among 3340 immunocompromised patients, mostly HIV/AIDS patients with diarrhea, whose prevalence ranged from 0% to 36% [11]. In another study, the prevalence rate of *Cyclospora* in 1088 stool samples from 544 symptomatic HIV positive cases was 2.2%, and no parasites were observed in asymptomatic cases [50]. Data accumulated in another systematic review of 14 sub-Saharan countries revealed an overall prevalence of *C. cayetanensis* of 18% [62].

It is important to note that the severity of disease caused by *C. cayetanensis* infection depends mainly on the host’s immune system. It is more severe in immunocompromised people, particularly PLWHA. A study carried out by Li et al. (2020) showed that the prevalence of *C. cayetanensis* was 7.38% (95% CI, 6.55–8.20%) in immunocompromised patients with diarrhea and 4.91% (95% CI, 4.35–5.47) in immunocompromised individuals without diarrhea. As a result, the reported prevalence of *C. cayetanensis* in this study was higher in people with diarrhea [63].

In our study, we showed that the highest and lowest prevalence rate among PLWHA was in North America and Asia, respectively. Cyclosporiasis is described in many countries, but it is most common in tropical and subtropical areas. It seems that the prevalence of this parasite is higher in underdeveloped and developing countries, and the effect of rainfall and weather on the prevalence of this parasite has already been shown [64]. We performed a meta-regression ratio for HIV and showed that the chances of contracting *C. cayetanensis* were 4.36 (95% CI, 2.00–9.48) in PLWHA.

We also found a significant relationship between *C. cayetanensis* infections and HIV patients (*p* < 0.05). Therefore, HIV infection is a risk factor for contracting this parasite. Immunodeficiency, especially HIV, and low CD4 counts are acknowledged important risk factors [65]. We also performed a ratio of patients’ CD4 counts <200 cells/mL and showed that these people had a 4.07 (95% CI, 1.37–12.12) chance of developing *C. cayetanensis* and that there was a significant relationship between CD4 counts and parasite infection (*p* < 0.05). HIV during its acute phase has been shown to cause a rapid decrease in CD4 cells in the lymphoid tissues of the gastrointestinal tract in the small and large intestine. Immune cells play an important role in repairing and maintaining the epithelial junction of the intestinal mucosa, and their discharge leads to impairment [66]. These cells integrate the mucosa of the intestinal wall, which in turn increases the transfer of microbes from the lumen into the lamina propria [67]. The immunity of those infected with HIV may be somewhat increased by HAART. Therefore, we also examined the relationship between *C. cayetanensis* infection and HAART. Our study showed that HAART significantly reduced the risk of *C. cayetanensis* infection. However, due to the limited data available, we could not find a significant relationship between them (*p* > 0.05). We continued to examine gender in the infection, but also owing to the lack of data, we could not see a significant difference between men and women (*p* > 0.05).

In general, our meta-analysis had several limitations. In most articles, there was not enough information about patients, so it was a limitation that we may missed some eligible data. Most of the studies except four [7,19,20,21] used direct methods of stool detection and staining. Diagnosis staining techniques included modified Ziehl–Neelsen (acid-fast), Safranin, Auramine, Rhodamine, Kinyoun, Giemsa, and Trichrome. The sensitivity of detection varies markedly between these techniques, depending on the protocol used and whether the oocysts are positively stained or not. Fluorescence-based microscopy provides an alternative means of detection. Nucleic acid-based methods provide an enhanced diagnostic and analytical performance, allowing for the specific and genotypic detection and identification of *C. cayetanensis*. Improved specificity and sensitivity were made possible largely through the use of PCR [68,69]. While microscopic diagnosis for this parasite is unreliable, the growing use of molecular methods may help to overcome some of the current diagnosis and treatment flaws. Due to the high sensitivity and accuracy of molecular methods, their use leads to more reliable results. By consequence, the gathering of these data may contribute to further reports and to the research and development for improved therapeutics.

## 5. Conclusions

In conclusion, the high prevalence of *C. cayetanensis* among PLWHA observed in this review emphasizes the need to implement screening and prophylaxis tailored to the local context for PLWHA. Data demonstrate that HIV-seropositive patients with diarrhea, CD4 cell count <200 cells/mL, and no antiviral treatment have higher prevalence of *C. cayetanensis* infection than other groups. These patients should receive early treatment for the non-specific symptoms caused by various parasitic diseases. Importantly, over the long interval between the time of infection and the onset of symptoms, physicians should treat the early symptoms of *C. cayetanensis*, such as diarrhea, in immunocompromised patients.

## Figures and Tables

**Table 1:** Assessment of risk of bias and quality in included studies.

NO	First Author	Q1	Q2	Q3	Q4	Q5	Q6	Q7	Q8	Q9	Score
1	J.W. Pape	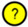	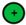	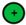	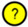	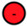	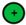	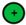	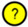	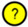	4
2	J. Sifuentes	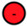	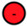	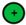	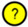	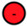	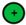	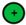	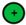	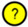	4
3	S. Manatsathit	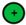	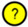	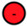	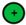	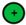	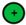	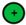	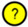	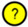	5
4	Y. Germani	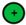	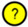	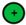	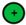	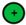	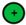	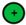	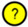	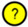	6
5	J.F. Lindo	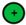	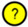	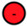	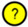	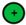	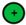	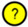	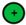	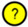	4
6	J.P. Cegielski	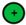	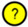	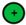	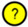	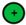	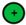	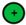	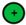	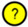	6
7	A. Escobedo	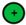	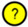	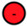	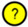	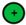	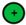	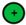	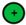	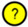	5
8	T. Gumbo	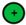	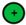	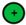	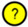	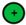	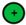	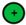	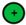	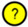	7
9	A. Mukhopadhya	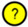	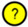	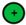	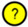	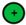	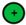	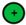	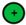	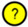	5
10	L. Chacin-Bonilla	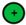	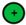	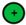	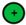	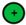	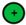	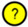	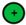	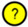	7
11	R.A. Pratdesaba	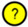	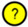	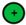	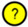	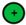	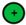	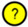	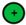	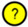	4
12	S.S. Kumar	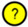	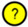	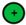	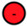	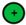	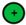	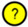	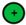	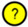	4
13	K. Mohandas	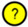	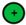	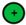	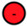	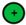	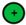	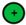	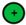	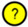	6
14	K.-X. Wang	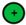	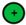	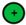	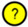	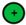	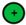	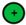	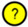	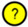	6
15	V. Capó de Paz	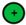	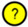	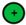	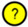	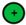	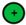	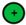	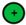	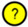	6
16	L. Chacin-Bonilla	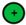	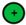	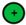	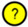	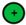	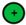	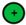	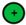	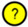	7
17	C. Sarfati	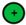	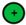	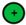	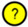	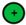	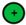	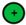	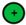	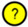	7
18	M.L. Becker	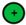	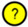	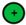	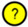	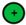	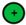	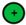	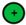	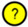	6
19	R.G. Kaminsky	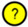	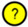	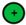	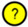	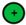	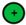	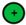	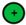	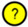	5
20	S. Gupta	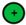	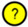	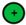	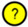	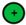	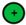	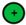	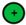	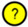	6
21	C.P. Raccurt	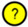	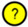	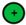	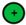	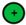	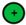	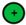	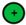	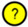	6
22	L. Tuli	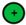	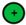	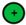	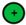	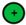	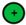	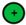	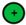	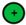	9
23	S. Kulkarni	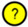	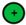	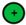	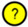	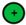	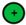	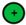	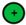	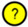	6
24	A. Kurniawan	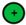	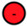	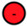	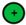	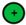	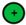	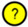	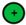	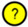	5
25	W. Saksirisampant	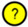	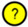	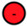	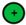	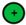	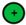	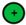	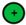	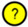	5
26	P. Viriyavejakul	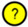	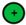	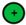	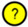	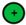	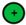	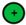	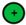	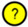	6
27	S. Babatunde	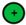	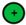	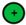	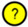	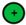	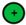	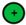	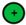	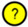	7
28	I. Asma	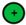	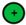	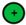	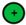	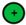	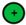	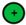	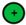	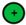	9
29	M. Agholi	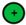	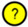	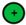	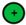	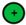	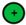	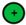	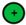	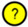	7
30	M.K. Mathur	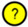	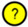	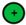	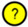	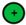	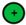	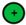	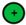	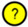	5
31	Z. Rivero-Rodríguez	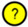	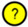	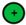	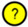	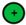	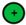	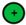	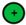	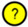	5
32	B.R. Tiwari	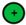	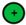	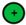	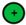	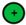	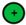	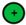	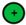	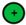	9
33	N.H. Ahmed	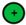	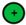	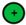	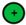	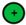	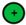	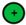	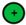	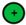	9
34	D.S. Nsagha	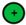	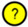	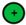	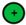	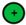	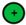	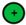	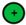	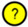	7
35	M. Agholi	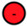	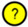	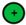	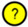	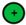	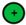	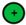	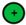	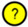	5
36	S. Shah	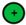	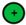	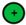	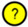	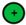	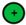	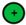	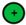	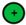	8
37	C.R. Swathirajan	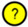	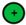	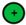	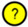	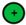	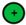	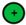	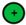	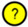	6
38	H.K. Uysal	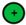	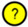	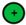	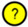	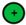	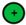	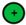	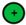	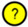	6
39	G. Alemu	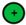	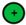	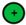	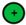	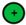	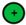	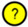	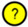	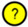	6
40	Y.K. Opoku	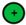	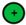	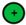	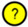	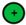	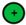	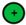	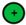	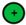	8
41	O. Zorbozan	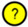	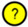	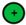	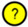	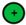	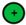	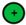	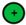	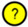	5
42	H. Masoumi-Asl	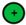	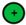	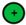	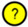	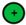	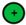	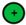	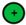	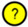	7
43	E.G. Rodríguez-Pérez	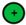	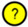	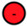	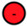	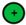	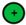	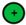	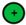	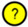	5
44	E.O. Udeh	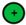	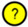	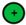	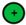	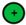	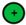	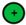	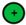	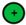	8
45	M.A.A.S. Namaji	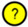	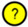	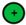	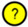	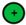	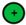	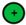	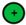	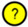	5

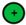
 YES. 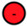 No. 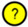 Unclear/Not applicable.

**Table 2:** Included studies of *C. cayetanensis* in people with HIV.

NO	First Author	Year	Country	Study Design	No. of Participants/Positive Patients	Mean Age	Diagnostic Method	Patients with Diarrhea	Patients with CD4 <200	Ref.
1	J.W. Pape	1994	Haiti	Cohort	450/43	24	Modified Kinyoun acid-fast	450	NR	[22]
2	J. Sifuentes	1995	Mexico City	Cross-sectional	235/7	40.5	Modified acid-fast	235	NR	[23]
3	S. Manatsathit	1996	Thailand	Prospective study	45/1	35	Modified acid-fast	45	45	[24]
4	Y. Germani	1998	Central African	Case-control	171/5	NR	Modified trichrome	171	171	[25]
5	J.F. Lindo	1998	Honduras	Cross-sectional	52/1	33.8	Modified Kinyoun acid-fast	16	NR	[26]
6	J.P. Cegielski	1999	Tanzania	Case-control	124/1	NR	Kinyoun and auramine rhodamine	124	NR	[27]
7	A. Escobedo	1999	Cuba	Case-control	67/2	27.9	Modified Ziehl–Neelsen	NR	NR	[28]
8	T. Gumbo	1999	Zimbabwe	Prospective study	88/0	33	Modified acid-fast	88	NR	[29]
9	A. Mukhopadhya	1999	India	Cross-sectional	111/5	NR	Safranine-Methylene blue and auramine	61	NR	[30]
10	L. Chacin-Bonilla	2001	Venezuela	Retrospective study	71/7	33.5	Modified Ziehl–Neelsen	71	NR	[31]
11	R.A. Pratdesaba	2001	Guatemala	Cross-sectional	157/6	32	Modified acid-fast	NR	NR	[32]
12	S.S. Kumar	2002	India	Case-control	152/1	40	Modified Kinyoun acid- fast and Safranin Methylene blue	102	NR	[33]
13	K. Mohandas	2002	Northern India	Cross-sectional	120/4	NR	Ziehl–Neelsen	26	NR	[34]
14	K.-X. Wang	2002	China	Case-control	32/3	NR	Auramine phenol stain and modified acid-fast	32	NR	[35]
15	V. Capó de Paz	2003	Cuba	Cross-sectional	170/6	NR	Ziehl–Neelsen	170	NR	[36]
16	L. Chacin-Bonilla	2006	Venezuela	Case-control	74/6	37.3 ± 5.6	Modified Ziehl–Neelsen	74	NR	[37]
17	C. Sarfati	2006	Cameroon	Cross-sectional	154/0	36	Modified Ziehl–Neelsen	46	NR	[38]
18	M.L. Becker	2007	India	Case-control	153/0	32.7 ± 8	Acid-fast trichrome	153	NR	[39]
19	R.G. Kaminsky	2007	Honduras	Cross-sectional	56/2	32.3	Acid-fast trichrome	18	NR	[40]
20	S. Gupta	2008	India	Cross-sectional	113/1	33.2 ± 9.72	Modified acid-fast	34	NR	[41]
21	C.P. Raccurt	2008	Haiti	Cross-sectional	67/27	NR	Weber modified trichrome	67	NR	[42]
22	L. Tuli	2008	India	Case-control	366/88	35.5	Modified acid-fast and modified safranin	366	236	[43]
23	S. Kulkarni	2009	India	Cross-sectional	137/1	M: 34.6 ± 7.51 F: 33.2 ± 9.95	Modified acid-fast	137	65	[44]
24	A. Kurniawan	2009	Indonesia	Cross-sectional	318/12	NR	Modified acid-fast	NR	NR	[45]
25	W. Saksirisampant	2009	Thailand	Cross-sectional	90/1	39.5	Modified Ziehl–Neelsen	71	NR	[46]
26	P. Viriyavejakul	2009	Thailand	Cross-sectional	64/3	NR	Ziehl–Neelsen	64	NR	[47]
27	S. Babatunde	2010	Nigeria	Cross-sectional	90/16	35	Modified Ziehl–Neelsen	90	26	[48]
28	I. Asma	2011	Malaysia	Cross-sectional	346/17	21.5	Modified Ziehl–Neelsen	30	189	[49]
29	M. Agholi	2013	Iran	Cross-sectional	356/1	37.18	Acid-fast trichrome stain, nested PCR	103	188	[19]
30	M.K. Mathur	2013	India	Retrospective study	544/12	42.5	Modified Ziehl–Neelsen	400	NR	[50]
31	Z. Rivero-Rodríguez	2013	Venezuela	Cross-sectional	56/2	35 ± 11.95	Modified Kinyoun acid-fast	48	NR	[51]
32	B.R. Tiwari	2013	Nepal	Cross-sectional	745/14	30	Modified acid-fast	248	327	[52]
33	N.H. Ahmed	2015	India	Cohort	142/3	NR	Modified Ziehl–Neelsen’s cold staining	142	NR	[53]
34	D.S. Nsagha	2015	Cameroon	Cross-sectional	300/11	40	Modified Ziehl–Neelsen	118	76	[54]
35	M. Agholi	2016	Iran	Cross-sectional	387/2	NR	Modified acid-fast or acid-fast trichrome and semi-nested PCR	387	NR	[7]
36	S. Shah	2016	India	Cross-sectional	45/2	34.5	Modified Ziehl–Neelsen	27	22	[55]
37	C.R. Swathirajan	2017	South India	Cross-sectional	829/2	M: 38 F: 33.5	Modified acid-fast	829	NR	[56]
38	H.K. Uysal	2017	Turkey	Cross-sectional	115/3	41.5	Ziehl–Neelsen and Kinyoun acid-fast, molecular methods	NR	11	[20]
39	G. Alemu	2018	Ethiopia	Cross-sectional	220/13	NR	Modified Ziehl–Neelsen	21	43	[57]
40	Y.K. Opoku	2018	Ghana	Cross-sectional	50/14	NR	Ziehl–Neelsen	50	NR	[8]
41	O. Zorbozan	2018	Turkey	Prospective study	65/2	41.9 ± 12.4	Modified acid-fast, Giemsa, Kinyoun	65	NR	[58]
42	H. Masoumi-Asl	2019	Iran	Cross-sectional	102/1	NR	Acid-fast and nested PCR	NR	9	[21]
43	E.G. Rodríguez-Pérez	2019	Mexico	Prospective study	29/0	37	Modified Ziehl–Neelsen, Giemsa and acid-fast trichrome	NR	NR	[59]
44	E.O. Udeh	2019	Nigeria	Case-control	891/9	NR	Ziehl–Neelsen	NR	3	[60]
45	M. Namaji	2020	India	Cross-sectional	361/7	NR	Modified acid-fast	361	NR	[61]

**Table 3:** Risk factors associated with *C. cayetanensis* infection in HIV patients.

Risk Factors	No. of Studies	Categories	OR (95% CI)	*p*-Value	I^2^ (Inconsistency) %	Cochran Q	*p*-Value
**Sex**	3	Male Female	1.72 (0.79–3.73)	*p* = 0.1647	0	2.06	*p* = 0.7244
**Diarrhea**	8	Yes No	3.23 (1.38–7.54)	*p* = 0.0066	0	4.81	*p* = 0.5675
**CD4**	10	<200 cells/mL >200 cells/mL	4.07 (1.37–12.12)	*p* = 0.0115	74.5	35.24	*p* < 0.0001
**HAART**	3	No Yes	2.07 (0.29–14.81)	*p* = 0.4668	-	1.57	*p* = 0.2101

## Data Availability

Not applicable.

## References

[1] Arora D., Arora B. (2009). AIDS-associated parasitic diarrhoea. Indian J. Med. Microbiol..

[2] Uzairue L.I., Oghena M., Ikede R.E., Aguda O.N., Adebisi Y.A., Lucero-Prisno D.E. (2021). Prevalence, risk factors and impact of cellular immunity on intestinal parasitosis among people living with HIV at Auchi, Edo State, Nigeria. Int. J. STD AIDS.

[3] UNAIDS DATA 2021. https://www.unaids.org/sites/default/files/media_asset/JC3032_AIDS_Data_book_2021_En.pdf.

[4] Batista F.S., Miranda L.d.S., Silva M.B.d.O., Taborda R.L.M., Soares M.C.F., Matos N.B. (2019). Chronic Cystoisospora belli infection in an HIV/AIDS patient treated at the specialized assistance service in Porto Velho County-Rondônia. Rev. Da Soc. Bras. De Med. Trop..

[5] Siddiqui U., Bini E.J., Chandarana K., Leong J., Ramsetty S., Schiliro D., Poles M. (2007). Prevalence and impact of diarrhea on health-related quality of life in HIV-infected patients in the era of highly active antiretroviral therapy. J. Clin. Gastroenterol..

[6] Dikman A.E., Schonfeld E., Srisarajivakul N.C., Poles M.A. (2015). Human immunodeficiency virus-associated diarrhea: Still an issue in the era of antiretroviral therapy. Dig. Dis. Sci..

[7] Agholi M., Shahabadi S.N., Motazedian M.H., Hatam G.R. (2016). Prevalence of enteric protozoan oocysts with special reference to Sarcocystis cruzi among fecal samples of diarrheic immunodeficient patients in Iran. Korean J. Parasitol..

[8] Opoku Y.K., Boampong J.N., Ayi I., Kwakye-Nuako G., Obiri-Yeboah D., Koranteng H., Ghartey-Kwansah G., Asare K.K. (2018). Socio-Behavioral Risk Factors Associated with Cryptosporidiosis in HIV/AIDS Patients Visiting the HIV Referral Clinic at Cape Coast Teaching Hospital, Ghana. Open AIDS J..

[9] Barazesh A., Fouladvand M., Tahmasebi R., Heydari A., Kooshesh F. (2016). Prevalence of intestinal parasitic infections among primary school children in Bushehr, Iran. Avicenna J. Clin. Microbiol. Infect..

[10] Saki J., Amraee D. (2017). Prevalence of intestinal parasites among the rural primary school students in the west of Ahvaz county, Iran, 2015. Jentashapir J. Health Res..

[11] Chacín-Bonilla L. (2010). Epidemiology of *Cyclospora cayetanensis*: A review focusing in endemic areas. Acta Trop..

[12] Wiwanitkit V. (2006). Intestinal parasite infestation in HIV infected patients. Curr. HIV Res..

[13] Naganathan T., O’Connor A., Sargeant J.M., Shapiro K., Totton S., Winder C., Greer A.L. (2022). The prevalence of *Cyclospora cayetanensis* in water: A systematic review and meta-analysis. Epidemiol. Infect..

[14] Dubey J.P., Almeria S., Mowery J., Fortes J. (2020). Endogenous developmental cycle of the human coccidian *Cyclospora cayetanensis*. J. Parasitol..

[15] Almeria S., Cinar H.N., Dubey J.P. (2019). *Cyclospora cayetanensis* and cyclosporiasis: An update. Microorganisms.

[16] Jiang H., Zhou Y., Tang W. (2020). Maintaining HIV care during the COVID-19 pandemic. Lancet HIV.

[17] Moher D., Shamseer L., Clarke M., Ghersi D., Liberati A., Petticrew M., Shekelle P., Stewart L.A. (2015). Preferred reporting items for systematic review and meta-analysis protocols (PRISMA-P) 2015 statement. Syst. Rev..

[18] Joanna Briggs Institute (2014). Joanna Briggs Institute Reviewers’ Manual 2014.

[19] Agholi M., Hatam G.R., Motazedian M.H. (2013). HIV/AIDS-associated opportunistic protozoal diarrhea. AIDS Res. Hum. Retrovir..

[20] Uysal H.K., Adas G.T., Atalik K., Altiparmak S., Akgul O., Saribas S., Gurcan M., Yuksel P., Yildirmak T., Kocazeybek B. (2017). The prevalence of *Cyclospora cayetanensis* and Cryptosporidium spp. in Turkish patients infected with HIV-1. Acta Parasitol..

[21] Masoumi-Asl H., Khanaliha K., Bokharaei-Salim F., Esteghamati A., Kalantari S., Hosseinyrad M. (2019). Enteric opportunistic infection and the impact of antiretroviral therapy among hiv/aids patients from Tehran, Iran. Iran. J. Public Health.

[22] Pape J.W., Verdier R.-I., Boncy M., Boncy J., Johnson W.D. (1994). Cyclospora infection in adults infected with HIV: Clinical manifestations, treatment, and prophylaxis. Ann. Intern. Med..

[23] Sifuentes-Osornio J., Porras-Cortés G., Bendall R.P., Morales-Villarreal F., Reyes-Terán G., Ruiz-Palacios G.M. (1995). *Cyclospora cayetanensis* infection in patients with and without AIDS: Biliary disease as another clinical manifestation. Clin. Infect. Dis..

[24] Manatsathit S., Tansupasawasdikul S., Wanachiwanawin D., Setawarin S., Suwanagool P., Prakasvejakit S., Leelakusolwong S., Eampokalap B., Kachintorn U. (1996). Causes of chronic diarrhea in patients with AIDS in Thailand: A prospective clinical and microbiological study. J. Gastroenterol..

[25] Germani Y., Minssart P., Vohito M., Yassibanda S., Glaziou P., Hocquet D., Berthelemy P., Morvan J. (1998). Etiologies of acute, persistent, and dysenteric diarrheas in adults in Bangui, Central African Republic, in relation to human immunodeficiency virus serostatus. Am. J. Trop. Med. Hyg..

[26] Lindo J.F., Dubon J.M., Ager A.L., De Gourville E.M., Solo-Gabriele H., Klaskala W.I., Baum M.K., Palmer C.J. (1998). Intestinal parasitic infections in human immunodeficiency virus (HIV)-positive and HIV-negative individuals in San Pedro Sula, Honduras. Am. J. Trop. Med. Hyg..

[27] Cegielski J.P., Ortega Y.R., McKee S., Madden J.F., Gaido L., Schwartz D.A., Manji K., Jorgensen A.F., Miller S.E., Pulipaka U.P. (1999). Cryptosporidium, Enterocytozoon, and Cyclospora infections in pediatric and adult patients with diarrhea in Tanzania. Clin. Infect. Dis..

[28] Escobedo A.A., Núñez F.A. (1999). Prevalence of intestinal parasites in Cuban acquired immunodeficiency syndrome (AIDS) patients. Acta Trop..

[29] Gumbo T., Sarbah S., Gangaidzo I.T., Ortega Y., Sterling C.R., Carville A., Tzipori S., Wiest P.M. (1999). Intestinal parasites in patients with diarrhea and human immunodeficiency virus infection in Zimbabwe. Aids.

[30] Mukhopadhya A., Ramakrishna B., Kang G., Pulimood A.B., Mathan M.M., Zachariah A., Mathai D.C. (1999). Enteric pathogens in southern Indian HIV-infected patients with & without diarrhoea. Indian J. Med. Res..

[31] Chacin-Bonilla L., Estévez J., Monsalve F., Quijada L. (2001). *Cyclospora cayetanensis* infections among diarrheal patients from Venezuela. Am. J. Trop. Med. Hyg..

[32] Pratdesaba R.A., González M., Piedrasanta E., Mérida C., Contreras K., Vela C., Culajay F., Flores L., Torres O. (2001). *Cyclospora cayetanensis* in three populations at risk in Guatemala. J. Clin. Microbiol..

[33] Kumar S.S., Ananthan S., Lakshmi P. (2002). Intestinal parasitic infection in HIV infected patients with diarrhoea in Chennai. Indian J. Med. Microbiol..

[34] Mohandas K., Sehgal R., Sud A., Malla N. (2002). Prevalence of intestinal parasitic pathogens in HIV-seropositive individuals in Northern India. Jpn. J. Infect. Dis..

[35] Wang K.-X., Li C.-P., Wang J., Tian Y. (2002). *Cyclospora cayetanensis* in Anhui, China. World J. Gastroenterol..

[36] Capó de Paz V., Barrero Brínguez M., Velázquez Viamonte B., Luzardo Suárez C., Martínez Rodríguez A., Alujas Martínez Z. (2003). Diagnóstico de coccidias y microsporas en muestras de heces diarreicas de pacientes cubanos seropositivos al VIH: Primer reporte de microsporas en Cuba. Rev. Cuba. De Med. Trop..

[37] Chacin-Bonilla L., Panunzio A.P., Monsalve-Castillo F.M., Parra-Cepeda I.E., Martinez R. (2006). Microsporidiosis in Venezuela: Prevalence of intestinal microsporidiosis and its contribution to diarrhea in a group of Human Immunodeficiency Virus–infected patients from Zulia State. Am. J. Trop. Med. Hyg..

[38] Sarfati C., Bourgeois A., Menotti J., Liegeois F., Moyou-Somo R., Delaporte E., Derouin F., Ngole E.M., Molina J.-M. (2006). Prevalence of intestinal parasites including microsporidia in human immunodeficiency virus–infected adults in Cameroon: A cross-sectional study. Am. J. Trop. Med. Hyg..

[39] Becker M.L., Cohen C.R., Cheang M., Washington R.G., Blanchard J.F., Moses S. (2007). Diarrheal disease among HIV-infected adults in Karnataka, India: Evaluation of risk factors and etiology. Am. J. Trop. Med. Hyg..

[40] Kaminsky R.G., Stovall M., Mayer M., Martin A.D., Bowers L.C., Didier E.S. (2007). Microsporidia intestinales en pacientes viviendo con SIDA en Honduras. Rev Méd Hondur.

[41] Gupta S., Narang S., Nunavath V., Singh S. (2008). Chronic diarrhoea in HIV patients: Prevalence of coccidian parasites. Indian J. Med. Microbiol..

[42] Raccurt C.P., Fouché B., Agnamey P., Menotti J., Chouaki T., Totet A., Pape J.W. (2008). Presence of Enterocytozoon bieneusi associated with intestinal coccidia in patients with chronic diarrhea visiting an HIV center in Haiti. Am. J. Trop. Med. Hyg..

[43] Tuli L., Gulati A.K., Sundar S., Mohapatra T.M. (2008). Correlation between CD4 counts of HIV patients and enteric protozoan in different seasons–An experience of a tertiary care hospital in Varanasi (India). BMC Gastroenterol..

[44] Kulkarni S., Kairon R., Sane S., Padmawar P., Kale V., Thakar M., Mehendale S., Risbud A. (2009). Opportunistic parasitic infections in HIV/AIDS patients presenting with diarrhoea by the level of immunesuppression. Indian J. Med. Res..

[45] Kurniawan A., Karyadi T., Dwintasari S., Sari I.P., Yunihastuti E., Djauzi S., Smith H. (2009). Intestinal parasitic infections in HIV/AIDS patients presenting with diarrhoea in Jakarta, Indonesia. Trans. R. Soc. Trop. Med. Hyg..

[46] Saksirisampant W., Prownebon J., Saksirisampant P., Mungthin M., Siripatanapipong S., Leelayoova S. (2009). Intestinal parasitic infections: Prevalences in HIV/AIDS patients in a Thai AIDS-care centre. Ann. Trop. Med. Parasitol..

[47] Viriyavejakul P., Nintasen R., Punsawad C., Chaisri U., Punpoowong B., Riganti M. (2009). High prevalence of Microsporidium infection in HIV-infected patients. Southeast Asian J. Trop. Med. Public Health.

[48] Babatunde S., Salami A., Fabiyi J., Agbede O., Desalu O. (2010). Prevalence of intestinal parasitic infestation in HIV seropositive and seronegative patients in Ilorin, Nigeria. Ann. Afr. Med..

[49] Asma I., Johari S., Sim B.L.H., Lim Y.A.L. (2011). How common is intestinal parasitism in HIV-infected patients in Malaysia?. Trop. Biomed..

[50] Mathur M.K., Verma A.K., Makwana G.E., Sinha M. (2013). Study of opportunistic intestinal parasitic infections in human immunodeficiency virus/acquired immunodeficiency syndrome patients. J. Glob. Infect. Dis..

[51] Rivero-Rodríguez Z., Hernández A., Bracho Á., Salazar S., Villalobos R. (2013). Prevalence of intestinal microsporidia and other intestinal parasites in hiv positive patients from Maracaibo, Venezuela. Biomedica.

[52] Tiwari B.R., Ghimire P., Malla S., Sharma B., Karki S. (2013). Intestinal parasitic infection among the HIV-infected patients in Nepal. J. Infect. Dev. Ctries..

[53] Ahmed N.H., Chowdhary A. (2015). Pattern of co-infection by enteric pathogenic parasites among HIV sero-positive individuals in a Tertiary Care Hospital, Mumbai, India. Indian J. Sex. Transm. Dis. AIDS.

[54] Nsagha D.S., Njunda A.L., Assob N.J.C., Ayima C.W., Tanue E.A., Kwenti T.E. (2015). Intestinal parasitic infections in relation to CD4+ T cell counts and diarrhea in HIV/AIDS patients with or without antiretroviral therapy in Cameroon. BMC Infect. Dis..

[55] Shah S., Kongre V., Kumar V., Bharadwaj R. (2016). A study of parasitic and bacterial pathogens associated with diarrhea in HIV-positive patients. Cureus.

[56] Swathirajan C.R., Vignesh R., Pradeep A., Solomon S.S., Solomon S., Balakrishnan P. (2017). Occurrence of enteric parasitic infections among HIV-infected individuals and its relation to CD4 T-cell counts with a special emphasis on coccidian parasites at a tertiary care centre in South India. Indian J. Med. Microbiol..

[57] Alemu G., Alelign D., Abossie A. (2018). Prevalence of opportunistic intestinal parasites and associated factors among HIV patients while receiving ART at Arba Minch Hospital in southern Ethiopia: A cross-sectional study. Ethiop. J. Health Sci..

[58] Zorbozan O., Quliyeva G., Tunalı V., Özbilgin A., Turgay N., Gökengin A.D. (2018). HIV ile Enfekte Olguların Bağırsak Protozoonları Açısından Retrospektif Olarak İncelenmesi. Turk. Parazitol Derg.

[59] Rodríguez-Pérez E.G., Arce-Mendoza A.Y., Montes-Zapata É.I., Limón A., Rodríguez L.É., Escandón-Vargas K. (2019). Opportunistic intestinal parasites in immunocompromised patients from a tertiary hospital in Monterrey, Mexico. Le Infez. Med. Riv. Period. Di Eziologia Epidemiol. Diagn. Clin. E Ter. Delle Patol. Infett..

[60] Udeh E.O., Obiezue R., Okafor F., Ikele C., Okoye I., Otuu C.A. (2019). Gastrointestinal parasitic infections and immunological status of HIV/AIDS coinfected individuals in Nigeria. Ann. Glob. Health.

[61] Namaji M.A.A.S., Pathan S.H., Balki A.M. (2020). Profile of intestinal parasitic infections in human immunodeficiency virus/acquired immunodeficiency syndrome patients in Northeast India. Indian J. Sex. Transm. Dis. AIDS.

[62] Chacín-Bonilla L. (2010). Importancia del contacto con la tierra en la transmisión de la ciclosporosis. Investig. Clínica.

[63] Burstein Alva S. (2005). Ciclosporosis: Una parasitosis emergente (II). Diagnóstico Microbiológico mediante una nueva técnica de coloración. Rev. De Gastroenterol. Del Perú.

[64] Fletcher S.M., Stark D., Ellis J. (2011). Prevalence of gastrointestinal pathogens in Sub-Saharan Africa: Systematic review and meta-analysis. J. Public Health Afr..

[65] Li J., Wang R., Chen Y., Xiao L., Zhang L. (2020). *Cyclospora cayetanensis* infection in humans: Biological characteristics, clinical features, epidemiology, detection method and treatment. Parasitology.

[66] Brenchley J.M., Douek D. (2008). HIV infection and the gastrointestinal immune system. Mucosal Immunol..

[67] Jagai J.S., Castronovo D.A., Monchak J., Naumova E.N. (2009). Seasonality of cryptosporidiosis: A meta-analysis approach. Environ. Res..

[68] Lobo M.L., Xiao L., Antunes F., Matos O. (2012). Microsporidia as emerging pathogens and the implication for public health: A 10-year study on HIV-positive and-negative patients. Int. J. Parasitol..

[69] Weinreich F., Hahn A., Eberhardt K.A., Feldt T., Sarfo F.S., Di Cristanziano V., Frickmann H., Loderstädt U. (2022). Comparison of Three Real-Time PCR Assays for the Detection of *Cyclospora cayetanensis* in Stool Samples Targeting the 18S rRNA Gene and the hsp70 Gene. Pathogens.

